# Robust dual sourcing inventory routing optimization for disaster relief

**DOI:** 10.1371/journal.pone.0284971

**Published:** 2023-04-27

**Authors:** Weibo Zheng, Hong Zhou

**Affiliations:** 1 School of Economics and Management, Beihang University, Beijing, China; 2 Beijing Key Laboratory of Emergency Support Simulation Technologies for City Operations, Beihang University, Beijing, China; HEC Montréal, CANADA

## Abstract

This paper considers the problem that a depot replenishes several shelters by aerial and land transportation modes for disaster relief. There are two distinguishing features of our problem: one is routing decisions determine replenishment lead times; the other is that we introduce dual sourcing policy into the inventory routing problem. A robust optimization model is proposed to determine the optimal replenishment quantity, replenishment mode, and transportation routes. Then, we decompose the problem into a routing master-problem and a set of inventory sub-problems. A tractable closed-form solution for sub-problem is derived. We further develop an adaptive large neighborhood search algorithm to solve the problem. To demonstrate the feasibility of the algorithm, we conduct a series of numerical experiments on the benchmark test suite with different scales and compare the performance of the proposed algorithm with a genetic algorithm.

## 1 Introduction

With the intensification of human activities, natural disasters, epidemics, and wars have led to many humanitarian disasters. Disasters lead to large-scale casualties, material shortages, and poor rescue environments. For example, an earthquake magnitude of 8.0 on the Richter scale struck Sichuan Province, China, on May 12, 2008 [1]. Thousands of damage incidents occur to roads, bridges, railways, and other basic infrastructures. In the early stages of relief operations, helicopters and trucks were used to deliver humanitarian supplies. In the critical first 72 hours, helicopters proved crucial in the affected area with poor road conditions. Multi-modal transport also minimizes the risk of emergency supplies failing to reach the affected area or breaking down emergency evacuation procedures [2].

Temporary shelters in the affected area play an essential role in disaster relief, that for accommodating patients and refugees, and providing food, water, and simple medical services. Governments and humanitarian organizations usually enhance their preparedness for disasters by pre-positioning inventory. However, storing large quantities of humanitarian relief supplies in shelters is unrealistic because of the poor storage conditions and the risk of secondary disaster. Emergency supplies are usually properly housed in one or more permanent or semi-permanent central depots. In emergency rescue, one crucial problem is delivering humanitarian relief from the central depot to shelters in the right amount and at the right time. However, structuring a post-disaster humanitarian relief distribution network to support emergency rescue for sudden-onset disasters is challenging because of demand uncertainty.

Due to secondary disasters and unpredictable accidents, shelters demand information in post-disaster areas is uncertain. It is difficult to deduce a specific distribution of demand. Robust optimization is a powerful approach to uncertainty problems. Compared to stochastic optimization, robust optimization doesn’t require knowledge of the distribution of uncertain information. Therefore, robust optimization is widely used in emergency rescue problems with uncertain demand. In addition, disasters like earthquakes lead to deteriorated transportation situations, such as road damage. It increases the difficulties of rescue. Road damage also results in a longer delivery time, even more than a few days when using land transportation. To ensure relief’s timeliness, combing aerial and land replenishment is an essential strategy in emergency logistics [3].

In emergency rescue, the inventory management and material deployment of shelters are usually taken over by a unified manager, such as the Red Cross organization. The joint optimization of inventory and routing mobilizes resources more timely and effectively to aid people affected by natural disasters and crises. Inventory routing problem (IRP) is first studied by Bell et al. (1983) under the context of vendor-managed inventory (VMI) [4]. Traditional IRP studies a distribution system including one supplier and several customers. The objective is to design an inventory policy and a set of vehicle routes to minimize the total cost of inventory and delivery. Traditional IRP assumes that the lead time is not impacted by route decisions. However, this assumption is not always reasonable, especially in disaster relief. The lead time for replenishment from the central depot to shelters is almost only composed of transportation. The different delivery routes affect the replenishment arrival time and, thus, the lead time of shelters. It links the inventory policy and the route decision much tighter and more complex.

This paper addresses a joint optimization problem of distributing humanitarian relief supplies from a central depot to several temporary shelters. Under the highly uncertain situation in post-disaster, planning a long-term policy is very difficult. We focus on a single-period inventory routing problem. A robust dual sourcing inventory routing problem (RDSIRP) model is proposed, which considers the impact of routing decisions on lead times. Dual-sourcing inventory policy uses two replenishment sources, where the regular one is cheaper but slower than the other one. We introduce the dual sourcing policy to IRP, which uses an aerial replenishment mode (usually by helicopter) and a land replenishment mode (usually by truck). For each shelter, there are four replenishment policies: the shelter is replenished by truck, by helicopter, by both, or by no replenishment. In this problem, three decisions are made: first, the selection of shelters to be replenished and the corresponding mode, second, the amount of delivered supplies, and third, the sequence of the shelters’ replenishment. They belong to three kinds of problems: assignment problems, inventory policies, and routing problems. We derive a closed-form solution of the optimal replenishment quantity under different lead times for the inventory policy. Then an adaptive large neighborhood search (ALNS) algorithm is developed to solve the assignment and routing problems.

The remainder of the paper is organized as follows: In section 2, the relevant investigations are reviewed and summarized. Section 3 describes the RDSIRP and presents mixed-integer programming formulations for the problem. In section 4, we derive a closed-form solution for the inventory policy and present our ALNS, followed by numerical experiment results in section 5 and our conclusions in section 6.

## 2 Literature review

Humanitarian logistics is essential because unavailability or slowness in mobilizing supplies causes increased human suffering and loss of life when a disaster strikes. Despite humanitarian logistics’ importance, the literature is limited in this area [5]. In recent years, humanitarian logistics has been studied in many aspects. Awan and Shafiq (2015) proposed a supply chain management framework for humanitarian logistics and explain how to overcome logistics difficulties during relief operations [6]. For the humanitarian relief inventory management aspect, Balcik et al. (2016) made a literature review on pre-disaster and post-disaster inventory management in humanitarian [7]. For the humanitarian relief delivery aspect, Holguín-Veras et al. (2013) proposed an exponential function to estimate the economic value of human suffering resulting from the lack of access to goods or service [8]. Then Çankaya et al. (2019) proposed an objective to minimize human suffering by minimizing the maximum length of the shortage duration [9]. Zhang et al. (2017) proposed a programming model for the vehicle routing problem (VRP), which combines air and ground transportation for emergency logistics [3]. Then Zheng and Zhou (2018) studied an air-ground transportation routing problem considering road damage in disasters [10]. Balcik and Yanıkoğlu (2020) studied a robust routing problem under travel time uncertainty in post-disaster, they used an evaluation function to choose which customer should be visited [11]. Yang et al. (2018) studied an inventory slack routing problem that applied to relief distributions [12]. Qiu et al. (2015) studied the time uncertainty in emergency logistics based on the grey theory optimization model [13].

In emergencies, the distribution information of demands is hard to estimate. Robust optimization is a powerful methodology for uncertain problems with unknown distribution. Soyster and Allen (1973) first studied robust optimization for solving linear programming problems with uncertain parameters, considering their worst case values under the uncertainty set [14]. The assumption that all uncertain parameters will get their worst case values is too conservative. To overcome the solution over-conservativeness, Ben-Tal and Nemirovski (1998) developed the ellipsoidal uncertainty set for uncertain convex optimization problems [15]. However, the ellipsoidal uncertainty set increases the problem’s complexity. Then Bertsimas and Thiele (2006) developed a robust optimization approach based on budget uncertainty set to inventory theory [16]. Based on the dual balance policy (see [17]), Mamani et al. (2017) derived a closed-form order size for robust inventory problem [18]. Then, Sun and van Mieghem (2019) provided a closed-form robust dual-sourcing policy for inventory problems by normalizing the slow ordering cost and fast lead time to 0 [19]. Ji et al. (2022) proposed three stochastic models to study uncertain factors and apply the L-shaped algorithm to achieve an optimal solution [20]. Qu et al. (2023) applied robust optimization to studying uncertainties in decision-making and propose four robust models based on the box, ellipsoidal, box-ellipsoidal, and box-polyhedral uncertainty sets, respectively [21].

The inventory routing problem is a joint optimization of inventory policies and routing decisions. It mobilizes resources timelier and more effectively, reducing human suffering and loss of life in emergencies. After the earlier works by Bell et al., Dror and Ball (1987) extended IRP to the multi-period finite horizon and proposed the first definition of the standard IRP [22]. Based on this framework, lots of variants based on the demand (deterministic or stochastic) and the planning horizon (finite or infinite) were studied. The stochastic demand IRP is a difficult challenge. Archetti et al. (2007) considered a finite planning horizon IRP with stochastic demand [23]. Hvattum et al. (2009) proposed a cyclic distribution strategy and points out that a finite scenario tree can capture most stochasticity, which means stochastic IRP can be solved by solving a scenario tree [24]. These IRP models did not consider transportation time consumption in lead times. Li et al. (2016) first studied a stochastic demand IRP with considering the replenishment routes affect lead times [25]. Then Zheng and Zhou (2019) presented a robust optimization model to deal with the uncertain demand and analyzed the influence between route decisions and order size [26]. Sanada et al. (2021) studied the inventory routing problem considering road closures in disaster [27].

Compared to other metaheuristics, the large neighborhood search (LNS) framework mainly employs insertion operators, which is very suitable for joint optimization of assignment and routing problems. The LNS framework was first introduced by Shaw (1998) and was applied to VRP with time windows [28]. It used a destroy-repair mechanism to search very large-scale neighborhoods. Shaw proposed a highly flexible removal operator called Shaw removal operator. In the LNS framework, operators can be highly customized by the problem. Then Røpke et al. (2006) proposed an adaptive large neighborhood search (ALNS) algorithm by introducing the adaptive mechanism to select removal and insertion operators [29]. Coelho et al. (2012) introduced the ALNS framework to IRP and developed a periodic post-optimization procedure to improve development ability [30]. Liu et al. (2020) studied a blood products inventory routing problem and proposed a decomposition-based algorithm [31]. The inventory sub-problem is solved by mixed-integer linear programming, and ALNS solves the routing sub-problem.

In general, current research on humanitarian logistics IRP is insufficient. IRP in an emergency context has many new challenges that need to be addressed. This paper is a novel attempt to consider transportation time as the lead time in inventory and routing joint optimization for emergency logistics.

## 3 Problem description and formulations

**Fig 1:**
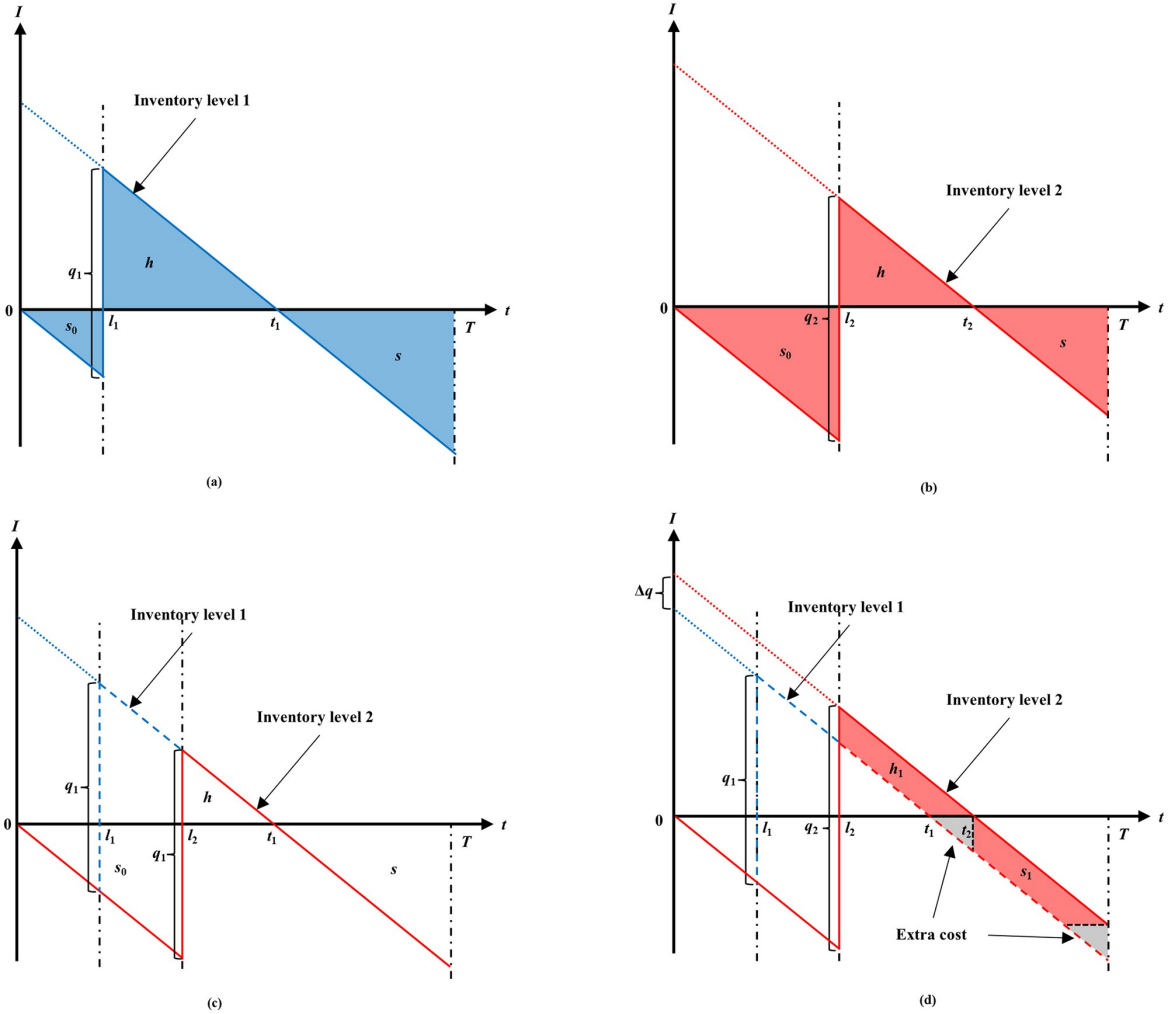
Later arrival time lead to a bigger replenishment quantity.

In post-disasters, several shelters are open for people in the affected area. The humanitarian relief central depot is responsible for choosing optimal routes and managing the inventory of the shelters. We consider the impact of delivery routes on the replenishment quantity in this paper. The following example illustrates why different arrival times lead to different replenishment quantities (as shown in Fig 1). For clarity and convenience, let us consider a simplified inventory problem and make the following assumptions: 1) the initial inventory level is 0; 2) the unit holding cost and the unit shortage cost are the same; 3) the demand per unit time is constant. The objective is to minimize the holding cost and the shortage cost by deciding the optimal replenishment quantity. *l*_1_ and *l*_2_ indicate two different possible lead times of a shelter. The replenishment quantity cannot impact the inventory cost before the replenishment arrival (*s*_0_). If we make the decision based on the lead time of *l*_1_, the optimal replenishment quantity would be *q*_1_, and the out-of-stock point is *t*_1_, where in the middle of *l*_1_ to *T*. As shown in sub-figure (a). Similarly, when we make the decision based on the lead time of *l*_2_, the optimal replenishment quantity would be *q*_2_, and the out-of-stock point is *t*_2_. As shown in sub-figure (b). Assuming *l*_1_ is the normal arrival time of replenishment, and *l*_2_ is the delayed arrival time. If the replenishment delay to *l*_2_ and still replenish *q*_1_, then the out-of-stock point is *t*_1_. It leads to a larger shortage cost and a smaller holding cost than the normal arrival time. As shown in sub-figure (c). The sub-figure (d) shows the extra cost of *q*_1_ than *q*_2_ when the replenishment is delayed. An extreme example is that if the supplies arrive after *t*_1_, and we still replenish *q*_1_ according to the lead time *l*_1_, then the shelter keeps out-of-stock throughout the planning horizon. Apparently, the later arrival time leads to a larger replenishment quantity. The larger replenishment quantity reduces inventory costs after replenishment arrival. But replenishment delays still incur higher inventory cost for the entire planning horizon.

### 3.1 Problem definitions

In this problem, we define a directed graph *G* = (*V*, *A*), where *V* = {0, …, *n*} is the vertex set and *A* = {(*i*, *j*) ∈ *V*, *i* ≠ *j*} is the arc set. Vertex 0 represents the depot and the vertexes of *V*′ = *V*∖{0} represent shelters. Arc (*i*, *j*) represents the shortest route between vertex *i* and vertex *j*. The planning horizon is *T*. The initial inventory level is revealed at the beginning of the planning horizon. Then, a truck and a helicopter depart from the central depot and return to the depot in the planning horizon. The helicopter’s unit time shipping cost is *f*^1^, and the truck’s unit time shipping cost is *f*^0^. where *f*^1^ > *f*^0^. *γ* represents replenishment modes, where *γ* ∈ {0, 1}, 0 represents the land replenishment mode, and 1 represents the aerial replenishment mode. We assume the lead time of each shelter in each replenishment mode is the time consumption of transport. The truck may arrive earlier than the helicopter for a shelter because of the delivery sequence. Define *l*^*f*^ = min {*l*^0^, *l*^1^} and *l*^*s*^ = max {*l*^0^, *l*^1^} as the lead time of the faster one and the lead time of the slower one, respectively. Similarly, *q*^*f*^ and *q*^*s*^ are the replenishment quantity of the faster one and the slower one, respectively.

Due to the affected area’s incomplete information, demands are highly uncertain. We assume the distribution of demands is unknown. Demands are independent, and values are in the positive symmetric interval $\left[{\bar{d}}_{it}-{\hat{d}}_{it},{\bar{d}}_{it}+{\hat{d}}_{it}\right]$, where ${\bar{d}}_{it}$ is the estimated demand of shelter *i* at time unit *t*, and ${\hat{d}}_{it}$ is the maximum deviation for the demand of shelter *i* at time unit *t*. At the time unit *t*, the demand of the shelter *i* is deducted from the total amount of the on-holding stock. If the total amount available is unmet demand, the unmet demand is backlogged to be satisfied in the future and causes a shortage cost. Otherwise, excess inventories are carried over at shelter *i* to satisfy future demands and cause a holding cost. Every shelter has a unit holding cost *h* and a unit shortage cost *s*, where *s* > *h*. When *s* ≫ *h*, out-of-stock will be avoided as much as possible.

**Fig 2:**
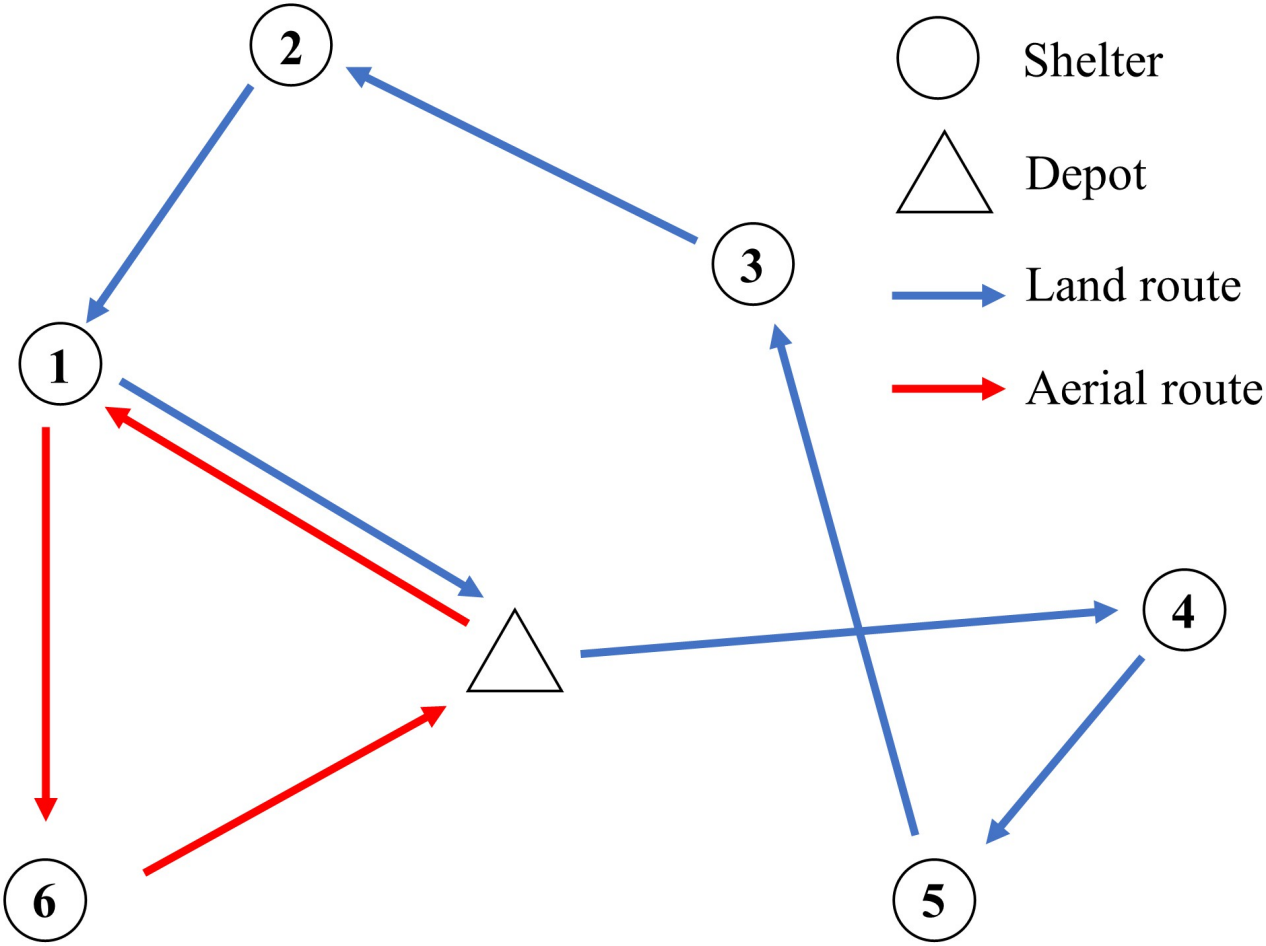
Illustration of the dual sourcing inventory routing problem.

To understand this problem intuitively, a set of possible delivery routes shows in Fig 2. Unlike traditional TSPs, each delivery route in this problem does not necessarily need to serve all shelters. Due to each shelter’s inventory cost, the optimal delivery route may not be the shortest.

### 3.2 Dual sourcing inventory routing model

An inventory routing model considering the replenishment lead time is proposed. Notations not mentioned in the problem definitions are listed as follows:

$c_{ij}^{\gamma }$ : The time cost of transportation from vertex *i* to vertex *j* by mode *γ*.

*M*: A big enough positive constant.

*I*_*i*_: The initial inventory level of shelter *i* at the beginning of the planning horizon.

*n*_*it*_: On-holding stock of shelter *i* at time *t*.

$x_{ij}^{\gamma }$ : 1 if the route of replenishment mode *γ* visits vertex *j* immediately from vertex *i*; 0 otherwise.

The objective function is the logistic cost within the planning horizon. The first item is the transportation cost of the two replenishment modes, and the second item is the inventory cost of all shelters.

$$\begin{array}{c} \text{min}:\;\sum_{\gamma \in \{0,1\}}\;f^{\gamma }\sum_{i\in V}\sum_{j\in V}c_{ij}^{\gamma }x_{ij}^{\gamma }+\sum_{i\in V^{\prime }}\sum_{t\in T}\;\text{max}\{hn_{it},-sn_{it}\}. \end{array}$$ (1)

$$\begin{array}{ccc} s.t.: & \; & n_{it}=I_{i}+\sum_{\gamma \in \{0,1\}}q_{i}^{\gamma }\text{sgn}\{t-l_{i}^{\gamma }\}-\sum_{\delta =1}^{t}{\tilde{d}}_{i\delta }\;\forall i\in V^{\prime },\forall t\in T, \end{array}$$ (2)

$$\begin{array}{ccc} & \; & q_{j}^{\gamma }\leq M\sum_{i\in V}x_{ij}^{\gamma }\;\forall j\in V^{\prime },\forall \gamma , \end{array}$$ (3)

$$\begin{array}{ccc} & \; & l_{i}^{\gamma }+c_{ij}^{\gamma }-\left(1-x_{ij}^{\gamma }\right)M\leq l_{j}^{\gamma }\;\forall i\in V,\forall j\in V^{\prime },i\neq j,\forall \gamma , \end{array}$$ (4)

$$\begin{array}{ccc} & \; & \sum_{i\in V}x_{ij}^{\gamma }\leq 1\;\forall j\in V,\forall \gamma , \end{array}$$ (5)

$$\begin{array}{ccc} & \; & \sum_{i\in V}x_{ij}^{\gamma }=\sum_{i\in V}x_{ji}^{\gamma }\;\forall j\in V,\forall \gamma , \end{array}$$ (6)

$$\begin{array}{ccc} & \; & \sum_{i\in V}\sum_{j\in V}c_{ij}^{\gamma }x_{ij}^{\gamma }\leq T\;\forall \gamma , \end{array}$$ (7)

$$\begin{array}{ccc} & \; & x_{ij}^{\gamma }\in \{0,1\}\;\forall i,j\in V,\forall \gamma , \end{array}$$ (8)

$$\begin{array}{ccc} & \; & 0\leq l_{i}^{\gamma }\leq T\;\forall i\in V^{\prime },\forall \gamma , \end{array}$$ (9)

$$\begin{array}{ccc} & \; & q_{i}^{\gamma }\geq 0\;\forall j\in V^{\prime },\forall \gamma . \end{array}$$ (10)

Constraint (2) is inventory balance equation, where $\sum_{\delta =1}^{t}d_{i\delta }$ is the cumulative demand of shelter *i* at time *t*. sgn{} is an indicator function, sgn {*A*} = 1 if *A* > 0, and sgn {*A*} = 0, otherwise; constraint (3) guaranteeing the replenishment quantity is zero if no replenishment mode service the customer *j*; constraint (4) is used to determine the arrival time which equals to the replenishment lead time, and it also eliminates subtours; constraint (5) ensures each shelter only be serviced no more than once; constraint (6) is the flow conservation constraint; constraint (7) ensures truck and helicopter back to the depot within the planning horizon; (8) and (9) are integer constraints; (10) is the non-negativity constraint.

**Fig 3:**
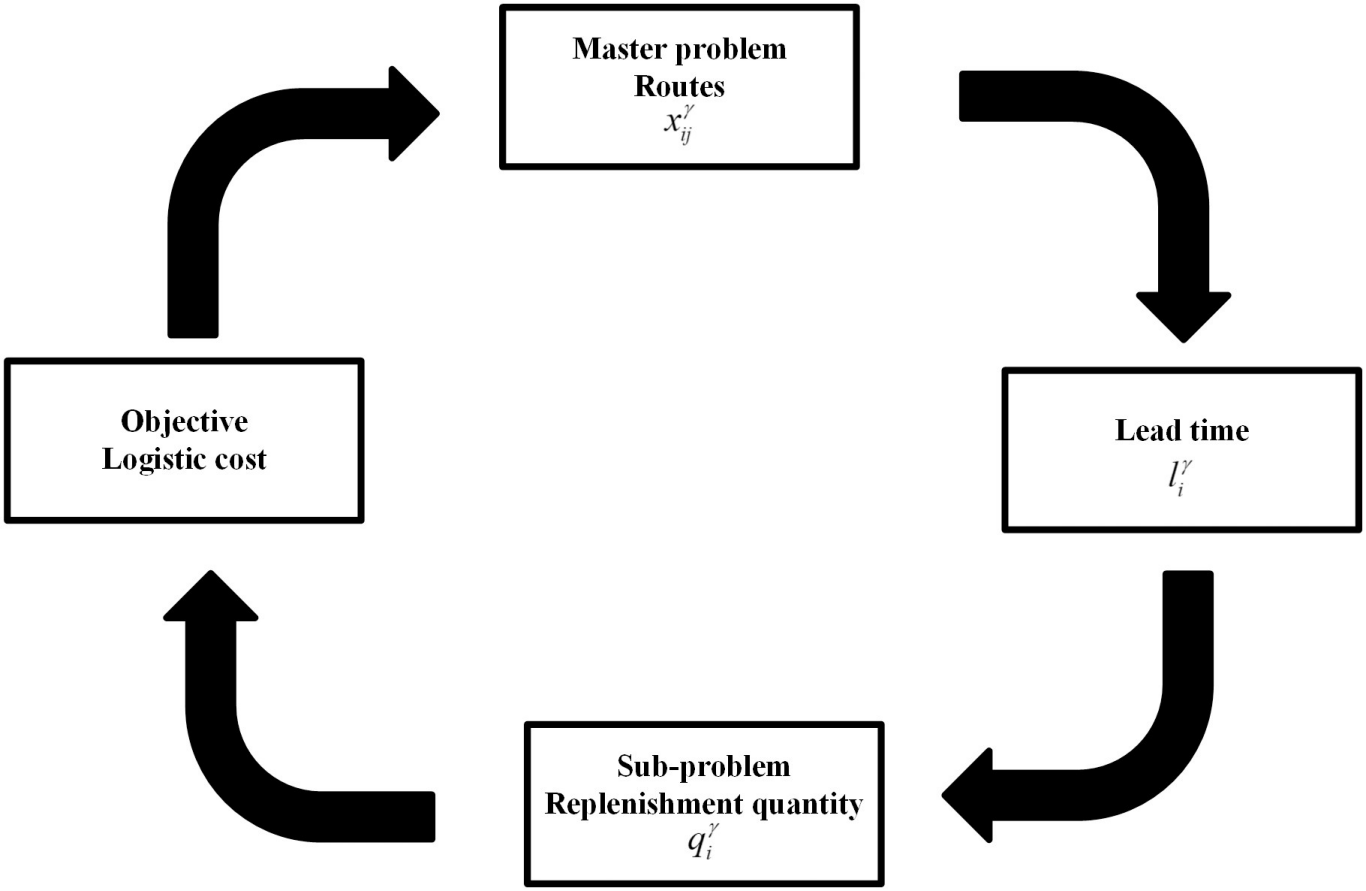
Interactions between the master-problem and sub-problems.

In constraint (2), $\text{sgn}\{t-l_{i}^{\gamma }\}$ is relevant to the variable $l_{i}^{\gamma }$, which means the inventory policy and the routing decision have a strong correlation by the lead time. So, we divide the model into the master-problem and a set of sub-problems. The master problem is a routing problem with constraints (3–10), considering inventory cost in the objective function. The sub-problem is an inventory problem with constraints (2, and 10), and the objective is to minimize the shortage cost and the holding cost. For the sub-problem, the lead time $l_{i}^{\gamma }$ depends on the routing problem constraints (4), which is an exogenous variable. The optimal inventory policy is to determine the replenishment quantity under the trade-off between holding cost and shortage cost. For the master problem, which route be chosen depends on the objective function value that includes the objective value of the sub-problem. Fig 3 shows the interaction between the master problem and sub-problems. The master problem determines the replenishment routes by the decision variable $x_{ij}^{\gamma }$. When the route is determined, the replenishment arrival time (lead time $l_{i}^{\gamma }$) of each shelter is revealed. Then the sub-problem determines the optimal replenishment quantities $q_{i}^{\gamma }$ based on lead times. The solution of the master problem and sub-problems determines the routing cost and the inventory cost. Then the master problem is searching for better replenishment routes to optimize the logistic cost.

### 3.3 Robust formulations

Given a set of replenishment routes, lead times are exogenous for sub-problems. The optimal inventory policy is to find an optimal replenishment quantity under the given lead time. The decision would be over conservativeness when we consider the worst case of the demand in the interval $\left[{\bar{d}}_{it}-{\hat{d}}_{it},{\bar{d}}_{it}+{\hat{d}}_{it}\right]$. A budget of uncertainty is imposed to ensure the total variation of the uncertainty parameters cannot exceed some threshold Γ. For the shelter *i*, we define the vector of future demands is contained in a non-negative bounded budget uncertainty set $U_{it}^{budg}$

$$\begin{array}{c} U_{it}^{budg}=\{d_{it}:\sum_{\delta =1}^{t}\frac{\left|d_{i\delta }-{\bar{d}}_{i\delta }\right|}{{\hat{d}}_{i\delta }}\leq \Gamma_{t},0\leq {\bar{d}}_{it}-{\hat{d}}_{it}\leq d_{it}\leq {\bar{d}}_{it}+{\hat{d}}_{it}\;\forall t\in T\}. \end{array}$$ (11)

where Γ_*t*_ is a non-decreasing threshold in *t* to control the total variation of demands under an assumption that uncertainty increases with the number of periods considered [16]. We define Γ_*t*_

$$\begin{array}{c} \Gamma_{t}=\text{min}\left(\sqrt{\frac{t}{1-\lambda^{2}}},t\right). \end{array}$$ (12)

Where λ = (*s* − *h*)/(*s* + *h*). For each shelter and any possible lead time, a sub-problem is formulated as follows:

$$\begin{array}{c} \begin{array}{cc} \text{min}: & \;\sum_{t\in T}\underset{d_{it}\in U_{it}^{budg}}{\text{max}}\;\{hn_{it},-sn_{it}\}\\ s.t.: & \;n_{it}=I_{i}+\sum_{\gamma \in \{0,1\}}q_{i}^{\gamma }\text{sgn}\{t-l_{i}^{\gamma }\}-\sum_{\delta =1}^{t}{\tilde{d}}_{i\delta }\;\forall t\in T\\ & \;q_{i}^{\gamma }\geq 0\;\forall \gamma \end{array} \end{array}$$ (13)

where $l_{i}^{\gamma }$ is a parameter determined by replenishment routes. We solve the robust optimization problem for each shelter that minimizes the worst case cost. To describe the worst case, we first define the minimum and maximum cumulative demands.

Assuming the cumulative demand is non-decreasing (for all *d*_*iδ*_ ≥ 0), for any uncertainty set *U*, the worst case of cumulative demand ${\underline{D}}_{it}$ and ${\bar{D}}_{it}$ can be denoted as ${\underline{D}}_{it}=\underset{(d_{i1},\cdots ,d_{it})\in U}{\text{min}}\;\sum_{\delta =1}^{t}d_{i\delta }$ and ${\bar{D}}_{it}=\underset{(d_{i1},\cdots ,d_{it})\in U}{\text{max}}\;\sum_{\delta =1}^{t}d_{i\delta }$, respectively. Under the budget uncertainty set $U_{it}^{budg}$, the worst case of cumulative demand is formulated as follows:

$$\begin{array}{c} \begin{array}{cc} {\underline{D}}_{it}=\text{min}: & \;\sum_{\delta =1}^{t}\left({\bar{d}}_{i\delta }-\omega_{i\delta }{\hat{d}}_{i\delta }\right)\\ s.t.: & \;\sum_{\delta =1}^{t}|\omega_{i\delta }|\leq \Gamma_{t}\\ & \;0\leq |\omega_{i\delta }|\leq 1\;\forall \delta \in \{1,\cdots ,t\} \end{array} \end{array}$$ (14)

and

$$\begin{array}{c} \begin{array}{cc} {\bar{D}}_{it}=\text{max}: & \;\sum_{\delta =1}^{t}\left({\bar{d}}_{i\delta }+\omega_{i\delta }{\hat{d}}_{i\delta }\right)\\ s.t.: & \;\sum_{\delta =1}^{t}|\omega_{i\delta }|\leq \Gamma_{t}\\ & \;0\leq |\omega_{i\delta }|\leq 1\;\forall \delta \in \{1,\cdots ,t\} \end{array} \end{array}$$ (15)

Where $\omega_{i\delta }=\left(d_{i\delta }-{\bar{d}}_{i\delta }\right)/{\hat{d}}_{i\delta }$.

We introduce the auxiliary variables *z*_*it*_, called the maximum mismatch (holding or shortage) cost of shelter *i* at time unit *t*. Then the robust dual sourcing (RDS) optimization sub-problem is formulated as follows:

$$\begin{array}{c} \begin{array}{cc} \underset{z\geq 0}{\text{min}}\;: & \;\sum_{t\in T}z_{it}\\ s.t.: & \;z_{it}\geq h\left(I_{i}+\sum_{\gamma \in \{0,1\}}q_{i}^{\gamma }\text{sgn}\{t-l_{i}^{\gamma }\}-{\underline{D}}_{it}\right)\;\forall t\in T,\forall d_{it}\in U_{it}^{budg}\\ & \;z_{it}\geq -s\left(I_{i}+\sum_{\gamma \in \{0,1\}}q_{i}^{\gamma }\text{sgn}\{t-l_{i}^{\gamma }\}-{\bar{D}}_{it}\right)\;\forall t\in T,\forall d_{it}\in U_{t}^{budg}\\ & \;q_{i}^{\gamma }\geq 0\;\forall \gamma \end{array} \end{array}$$ (16)

For any *I* and *l*, the robust optimization problem has an optimal solution, denoted by $q_{i}^{*}\left(I_{i},l_{i}^{\gamma }\right)=[q_{i}^{0*}\left(I_{i},l_{i}^{0}\right),q_{i}^{1*}\left(I_{i},l_{i}^{1}\right)]$. when the initial inventory level and the replenishment lead time are determined at the beginning of the planning horizon, the corresponding optimal inventory cost is

$$\begin{array}{c} C_{i}^{RDS*}=\sum_{t\in T}z_{it}\left({\text{q}}_{i}^{*}\right). \end{array}$$ (17)

## 4 Methodologies

In this section, we derive a closed-form robust optimal solution to the sub-problem. Then we develop an adaptive large neighborhood search algorithm to solve the master problem.

### 4.1 Closed-form sub-problem solution

During the iteration, RDS sub-problems need to be solved many times. A closed-form sub-problem solution would accelerate algorithms and make the inventory policy much easier to understand. The following proposition presents optimality conditions that RDS with exogenous lead time and initial inventory level.

**Proposition 1 (optimality conditions)** *For any uncertainty set U, and in any time unit t* = 1, ⋯, *T, since* $\left(h{\underline{D}}_{it}+s{\bar{D}}_{it}\right)/\left(h+s\right)$ *is non-negative and non-decreasing in t, the robust optimal replenishment quantity in the planning cycle of each mode satisfy*:

(1) *For any shelter replenished by single mode, the optimal replenishment quantity is*

$$\begin{array}{c} q_{i}^{*}=\text{max}\{\psi_{i}\left(t_{i}^{*}\right)-I_{i},0\}, \end{array}$$ (18)

*where* $t_{i}^{*}\in \{l_{i},\ldots ,T\}$, *and*

$$\begin{array}{c} \psi_{i}\left(t_{i}^{*}\right)=\frac{s{\bar{D}}_{{it}^{*}}+h{\underline{D}}_{{it}^{*}}}{h+s}. \end{array}$$ (19)

(2) *For any shelter replenished by both two modes, the optimal replenishment quantity of the faster arrival one is*

$$\begin{array}{c} q_{i}^{f*}=\text{max}\{\psi_{i}\left(t_{i}^{f*}\right)-I_{i},0\}, \end{array}$$ (20)

*and the slower one is*

$$\begin{array}{c} q_{i}^{s*}=\text{max}\{\psi_{i}\left(t_{i}^{s*}\right)-q_{i}^{f*}-I_{i},0\}, \end{array}$$ (21)

*where* $t_{i}^{f*}\in \{l_{i}^{f},\ldots ,l_{i}^{s}\}$ *and* $t_{i}^{s*}\in \{l_{i}^{s},\ldots ,T\}$, *respectively*.

The proof of Proposition 1 is in S1 Appendix. When a shelter is replenished by single mode, for out-of-stock point *t*_*i*_, we solve the optimal replenishment quantity by Eq (18). The point corresponding to the smallest inventory cost is $t_{i}^{*}$. When both two modes replenish a shelter, the optimal replenishment quantity *q*^f*^ and *q*^s*^ is solved by the combination of $t_{i}^{f}$ and $t_{i}^{s}$ by (20) and (21).

### 4.2 Adaptive large neighborhood search algorithm

**Fig 4:**
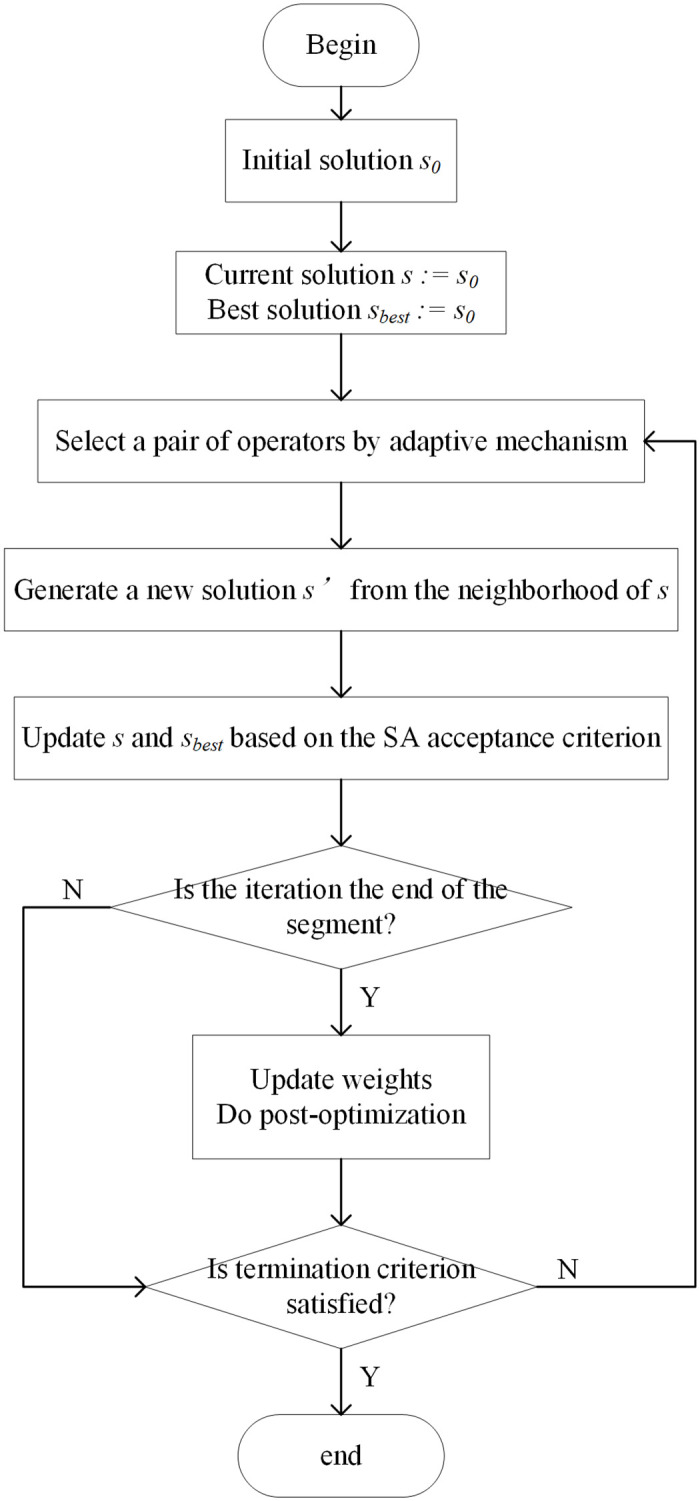
An illustration of the ALNS algorithm.

The problem we considered is NP-hard since the problem contains two TSPs. Developing a heuristic to deal with realistic size instances is necessary. The ALNS algorithm is suitable for the joint optimization of routing and assignment problems. It can handle the situation that only partial shelters are visited in a single replenishment mode. We describe our ALNS heuristic under the framework of [29, 30]: The algorithm begins with an initial solution *S*_0_, which is generated by the initial solution generator. One solution consists of two parts: the land replenishment mode and the aerial replenishment mode. Each replenishment mode corresponds to a route and a pool. Shelters not served by the replenishment mode will be placed in the reinsert pool. The roulette-wheel mechanism selects a pair of removal and insertion operators in each iteration and generates a neighborhood solution *S*′. The removal operator removes shelters from their current route, and the insertion operator inserts shelters into the way from the reinsert pool. The removal rate *ρ* controls the number of shelters removed by the removal operator in each iteration. The insertion operator inserts shelters into the route as much as possible until the logistic cost is no longer reduced. The current solution will be updated according to the simulated annealing (SA) mechanism which is based on the performance of *S*′. Search procedures are divided into several segments, and each segment contains *ξ* iterations. At the end of each segment, the selection probability of each operator is updated based on their performance in this segment, and we apply a 2-opt procedure to each mode as a periodic post-optimization. This process repeats until the termination criteria are satisfied. The framework is illustrated in Fig 4.

1. **Initial solution generator**
  The initial solution generator begins by adding all the shelters to reinsert pools, then alternately select a replenishment mode and adds the shelter with the lowest insertion cost to the end of the route until the insertion cost becomes positive. The insertion cost is defined as the difference in the objective value after inserting one shelter into the current route. Let *f*(*S*) represent the objective value of the current solution *S*, and *f*(*S*′) represent the objective value after the shelter *i* is inserted into a position of the route. The insertion cost for shelter *i* is *f*(*S*′) − *f*(*S*).
2. **Removal operators**
  **Shaw removal**: Shaw (1998) suggests that removing a set of shelters that are related is logical and useful [28]. We modified the operator to fit this problem. The relatedness between shelter *i* and *j* is described by *R*(*i*, *j*) as
  $$\begin{array}{c} R\left(i,j\right)=\varphi d_{ij}+\left(1-\varphi \right)|l_{i}^{*}-l_{j}^{*}|. \end{array}$$ (22)
  The relatedness measure consists of the distance *d* and the optimal replenishment lead time l*. We set the weight *φ* = 0.5. *d*_*ij*_ and $\left|l_{i}^{*}-l_{j}^{*}\right|$ are normalized. The smaller *R*(*i*, *j*) represents the higher relatedness. A shelter is randomly chosen from a removal pool of a replenishment mode. If the removal pool is empty, randomly remove a shelter in the route. Then the relatedness between a set of shelters in the current route and the already removed one is calculated. The highest relatedness shelter is removed from the current route. Check for the removed shelter. If both two replenishment modes replenish it, remove it from the route of another replenishment mode together. The complexity of this operator is *O*(*n*).
  **Distance removal**: Similar to the Shaw removal operator, the closest shelters are removed from the current route, where *φ* = 1. But the operator does not remove the same shelter in the route of another replenishment mode. The complexity is *O*(*n*).
  **Worst removal**: Let *f*(*S*) represents the objective value of the current solution *S*, and *f*(*S*′) represents the objective value after the shelter *i* removed from a route. The removed cost of shelter *i* is defined as *f*(*S*′) − *f*(*S*). This operator removes the shelter with the lowest removal cost, and the complexity is *O*(*n*).
  **Worst replenishment time removal**: If a shelter is replenished by one mode, we use replenishment time gap *g* to measure the gap between the arrival and optimal replenishment times. This operator removes the shelter with the highest *g*. The complexity of this operator is *O*(*n*).
  **Random removal**: This operator randomly removes a shelter from a route. The complexity of this operator is *O*(1).
3. **Insertion operators**
  **Greedy insertion**: The insertion cost for inserting each shelter in the removal pool into every feasible position is calculated. The insertion cost is defined as the same as the initial solution generator. Then insert the shelter with the minimum insertion cost to the insertion position. The complexity of this operator is *O*(*n*^2^)
  **2-regret insertion**: The regret insertion tries to use a kind of look-ahead information when selecting the shelter. For a replenishment mode *γ*, let $f_{ik}^{\gamma }$ be the insertion cost that the shelter *i* inserts the *k*’th position of the route. Then rank $f_{ik}^{\gamma }$ for every insert position *k*, $\Delta f_{i}^{\gamma }$ represents the regret value which is the difference in the cost of inserting the shelter in its best position and its second-best position. In each iteration, insert the shelter with the maximum regret value to the best insertion position accordingly, and the complexity is *O*(*n*^2^).
  **Random Shaw insertions**: First, put all shelters which not inserted any route into a candidate pool. Then select a shelter randomly from the candidate pool and calculate the relatedness between the chosen shelter and the others in the pool. Finally, a pair of shelters with the highest relatedness is selected and inserted into an adjacent position of a route with the lowest insertion cost. The complexity of this operator is *O*(*n*).
4. **SA acceptance criterion**
  We use an acceptance criterion based on the simulated annealing mechanism. Given a solution *S*, a neighbour solution *S*′ is accepted if *f*(*S*′) < *f*(*S*) and with probability *e*^−[*f*(*S*′)−*f*(*S*)]/*τ*^ otherwise, where *τ* > 0 is the current temperature. The temperature starts at *τ*_*start*_ and is decreased by a cooling rate factor *ϕ* at each iteration, where 0 < *ϕ* < 1.
5. **Adaptive mechanism**
  The choice of which operator to apply at an iteration is adapted by the roulette-wheel mechanism. Each operator has a weight and a score. The probability of each operator is based on its weight, depending on its past performance. Let *θ*_*i*_ is the weight of operator *i*, then operator *j* be selected with probability *θ*_*j*_/∑_∀*i*_ *θ*_*i*_. As mentioned before, the search progress is divided into segments of *ξ* iterations each. In each segment, initially, the score is equal to 0. At each iteration, if the neighbor solution *S*′ is better than the best solution, the score of operators used in the iteration is increased by *σ*_1_; if *S*′ is better than the current solution *S*, the score is increased by *σ*_2_; if *S*′ is accepted by the SA acceptance criterion, the score is increased by *σ*_3_; if *S*′ is not accepted, the score is increased by 0. After the last iteration of the current segment, update the weights of operators based on their score. We use *π*_*i*_ to represent the score of the operator *i* at the end of the current segment, and *κ*_*i*_ is the number of times has been used in the segment of the operator *i*. Then the weight in the next segment is updated by
  $$\begin{array}{c} \theta_{i}=\{\begin{array}{ccc} & \theta_{i}\; & \;\kappa_{i}=0\\ & \left(1-\eta \right)\theta_{i}+\eta \pi_{i}/\kappa_{i}\; & \kappa_{i}\neq 0 \end{array}, \end{array}$$ (23)
  where *η* = [0, 1] is called the reaction factor, controlling the adjustment speed.
6. **Periodic post-optimization**
  LNS mainly adopts insertion heuristics, which makes the development ability of this algorithm insufficient. In the improved heuristic, 2-opt performs well in inner route local search. We apply a 2-opt procedure at the end of each segment to optimize the current best solution. The 2-opt operation randomly selects two shelters in the route of a mode and then swaps the route between them. This operation repeats until the current best solution has no improvement times more than periodic iterations limits $Iter_{periodic}^{max}$, where the complexity is *O*(*n*) in the once 2-opt operation.
7. **Termination criteria**
  We limit the running time to 1h, maximum iterations *Iter*^*max*^ = 3000, and when the temperature is reached, the cut-off threshold *τ*_*cut*_ = 0.01.
8. **Parameter settings**
  We test different parameter combinations in a tuning phase to three randomly generated instances, including a small instance with 10 shelters, a middle instance with 50 shelters, and a large instance with 100 shelters, respectively. In addition, the selection of parameters also partially refers to the settings of [30]. The starting temperature *τ*_*start*_ is set to 30,000 and the cooling rate *ϕ* is 0.995. For large instances, more computing time per iteration is required, which leads to the temperature being too high when the time limit is reached. So, if the running time is more than 1/3 of the running time limit and the iterations are less than 1/3 maximum iterations, then the cooling rate is set to (*τ*_*cut*_/*τ*)^(1/*Iter*)^; if the running time is more than 2/3 of the running time limit and the iterations are less than 1/2 maximum iterations, then the cooling rate is set to (*τ*_*cut*_/*τ*)^(1/2*Iter*)^. This mechanism makes the temperature closer to *τ*_*cut*_ when the number of iterations is 3000 or the running time is 1 hour. The removal rate *ρ* = 20%. The segment capacity *ξ* = 50 and the reaction factor *η* = 0.2. Scores are updated with *σ*_1_ = 10, *σ*_2_ = 5, and *σ*_3_ = 2. The periodic iterations limits $Iter_{periodic}^{max}=200$.

## 5 Numerical experiments

This section provides the computational results related to our model solved by the ALNS. The ALNS is implemented on Python 3.9 and on a PC with Intel i9 10900 CPU @ 4.0GHz processor and 16 GB RAM. All numerical results reported in tables are the average of running five times. (The test set can be found at http://vrp.galgos.inf.puc-rio.br/index.php/en/. And the source code of the proposed ALNS algorithm can be found at https://github.com/WeberZheng/ALNS.)

### 5.1 Benchmark instances

There is no available benchmark instance in the literature that fits the problem we proposed. So, we adapt Augerat’s CVRP benchmark instance Set A and Set B to this problem. Node coordinates in the instance set A are generated in the range 100 × 100 randomly, and demands are generated in the interval [1, 30]. The node coordinates and demand information in the instance set B come from some practical application scenarios. The first node is the depot, and the other nodes are shelters. We use the demands information as the initial inventory level to fit our problem. We set the scale is 1:1km, the truck travels at a speed of 60km/h, and the helicopter travels at a speed of 260km/h. The typical trailer consumes 29.9 L/100km of fuel and the Sikorsky S-76C helicopter with 260km/h consumes 143 L/100 km. It is justifiable that we set the truck unit cost of use as 200 and the helicopter unit cost of use as 1000, which is similar to the fuel consumption ratio. We also set the unit holding cost as 2 and the unit shortage cost as 20. For convenience, we set all shelters to have the same predicted demand per unit time with $\bar{d}=4$. and $\hat{d}=2$. Considering the master problem is NP-hard, and the complexity of solving a sub-problem by the method we proposed is *O*(*T*). The planning horizon *T* to contain 8 time units to simulate 8 hours of working time and reduce the computational cost of experiments.

Under these instance parameter settings, these instances simulate two situations: The small-scale instances simulate the situation when the land replenishment mode can service all shelters within the time limit. The large-scale instances simulate the situation when the land replenishment mode cannot service all shelters within the planning horizon.

### 5.2 Model analysis

We first compare the logistic cost gaps of dual-sourcing to only land replenishment mode and only aerial replenishment mode. As shown in Table 1, the baseline of gaps is the dual-sourcing, where all Gaps in this paper are calculated by

$$\begin{array}{c} \text{Gap}=\frac{\text{Ob}{\text{j}}_{\text{other}}-\text{Ob}{\text{j}}_{\text{baseline}}}{\text{Ob}{\text{j}}_{\text{baseline}}}\times 100\%. \end{array}$$ (24)

**Table 1:** Logistic cost gaps between dual-sourcing and single replenishment modes.

Instance	Dual-sourcing	Only Truck		Only Helicopter	
	Obj	Obj	Gap	Obj	Gap
A-n32	13830.55	20680.15	49.53%	15049.80	8.82%
A-n33a	13929.27	18994.27	36.36%	15337.40	10.11%
A-n33b	14331.09	18006.74	25.65%	15887.21	10.86%
A-n34	14498.00	20906.73	44.20%	15971.69	10.16%
A-n36	15200.69	23563.32	55.01%	16624.04	9.36%
A-n37a	15915.37	27075.12	70.12%	17608.14	10.64%
A-n37b	16366.33	23850.18	45.73%	17680.50	8.03%
A-n38	15948.29	25326.41	58.80%	17605.10	10.39%
A-n39a	16806.88	28463.64	69.36%	18322.34	9.02%
A-n39b	17387.01	29742.34	71.06%	18643.53	7.23%
A-n44	18620.07	31857.34	71.09%	20148.66	8.21%
A-n45a	19133.85	35392.07	84.97%	20617.88	7.76%
A-n45b	18887.41	27969.92	48.09%	20476.51	8.41%
A-n46	19320.84	32127.82	66.29%	20860.87	7.97%
A-n48	20251.86	33627.27	66.05%	21642.41	6.87%
A-n53	22218.30	42313.86	90.45%	24409.52	9.86%
A-n54	22536.00	43083.44	91.18%	24280.00	7.74%
A-n55	23249.51	40412.33	73.82%	24666.17	6.09%
A-n60	25203.82	48630.00	92.95%	26775.05	6.23%
A-n61	25249.92	43591.08	72.64%	26961.10	6.78%
A-n62	25792.08	51956.62	101.44%	28001.32	8.57%
A-n63a	26103.63	45283.56	73.48%	28007.06	7.29%
A-n63b	25760.94	49717.94	93.00%	27768.16	7.79%
A-n64	26514.35	54070.95	103.93%	28445.89	7.28%
A-n65	26380.00	55618.02	110.83%	28557.17	8.25%
A-n69	28476.25	65905.74	131.44%	30769.43	8.05%
A-n80	32616.40	82275.51	152.25%	35382.40	8.48%
B-n31	11815.13	13389.95	13.33%	14133.60	19.62%
B-n34	14300.57	17466.92	22.14%	16745.55	17.10%
B-n35	14336.08	18381.76	28.22%	16449.62	14.74%
B-n38	14844.98	18515.91	24.73%	17375.05	17.04%
B-n39	14941.74	19623.89	31.34%	17638.49	18.05%
B-n41	15996.34	19492.17	21.85%	18207.22	13.82%
B-n43	16471.69	20155.06	22.36%	19082.90	15.85%
B-n44	17401.27	20867.43	19.92%	20216.32	16.18%
B-n45a	18178.73	30040.79	65.25%	20659.33	13.65%
B-n45b	16846.26	19892.90	18.08%	19755.17	17.27%
B-n50a	19888.49	27220.90	36.87%	22820.89	14.74%
B-n50b	19857.79	24760.11	24.69%	22734.49	14.49%
B-n51	20813.25	29989.25	44.09%	22736.16	9.24%
B-n52	19794.28	28861.21	45.81%	22907.44	15.73%
B-n56	21057.67	31408.23	49.15%	24372.43	15.74%
B-n57a	22323.83	34807.16	55.92%	25626.12	14.79%
B-n57b	21864.39	31897.35	45.89%	24967.39	14.19%
B-n63	24619.95	36220.14	47.12%	27076.65	9.98%
B-n64	24225.35	32028.25	32.21%	27823.53	14.85%
B-n66	24593.59	35485.08	44.29%	28156.51	14.49%
B-n67	25588.89	38217.25	49.35%	28886.33	12.89%
B-n68	25654.70	39075.71	52.31%	29467.00	14.86%
B-n78	30695.47	55145.26	79.65%	33804.21	10.13%
Average	-	-	58.49%	-	11.31%

The results show that the dual sourcing policy significantly reduces costs than only one replenishment mode policy. Only the land replenishment mode performs significantly poorly because only using the truck exceeds the planning horizon on large-scale instances. Moreover, in the case of only using the land replenishment mode, many shelters cannot be replenished in the planning horizon, leading to a higher inventory cost than the other two situations.

In addition, we compare the logistic costs between routing decisions with and without considering inventory cost (IC) under only one replenishment mode, and the baseline is routing considering inventory cost. The dual replenishment mode problem without considering inventory cost is not studied because the replenishment mode selection is highly related to inventory cost. For the routing solution without considering inventory cost, the shortest route is found by solving the mixed-integer linear programming of TSP. Gaps are reported in Table 2. The logistic cost considering the inventory cost is better than without considering the inventory cost when making the routing decision, where gaps are 21.04% and 4.98%, respectively.

**Table 2:** Logistic cost gaps between routing with/without considering inventory cost.

Instance	Only Truck			Only Helicopter		
	With IC	Without IC	Gap	With IC	Without IC	Gap
A-n32	20680.15	23922.25	15.68%	15049.80	15668.64	4.11%
A-n33a	18994.27	22791.73	19.99%	15337.40	15693.90	2.32%
A-n33b	18006.74	20382.81	13.20%	15887.21	16322.90	2.74%
A-n34	20906.73	22702.64	8.59%	15971.69	16435.83	2.91%
A-n36	23563.32	28025.67	18.94%	16624.04	17467.06	5.07%
A-n37a	27075.12	29893.08	10.41%	17608.14	17911.75	1.72%
A-n37b	23850.18	27992.52	17.37%	17680.50	18222.37	3.06%
A-n38	25326.41	28936.25	14.25%	17605.10	18103.83	2.83%
A-n39a	28463.64	40727.24	43.09%	18322.34	18537.12	1.17%
A-n39b	29742.34	36097.70	21.37%	18643.53	19236.88	3.18%
A-n44	31857.34	42556.08	33.58%	20148.66	21047.50	4.46%
A-n45a	35392.07	39499.34	11.61%	20617.88	21388.96	3.74%
A-n45b	27969.92	39276.39	40.42%	20476.51	21277.05	3.91%
A-n46	32127.82	39393.72	22.62%	20860.87	21895.06	4.96%
A-n48	33627.27	44450.82	32.19%	21642.41	22516.41	4.04%
A-n53	42313.86	55286.59	30.66%	24409.52	25475.84	4.37%
A-n54	43083.44	50859.37	18.05%	24280.00	25517.56	5.10%
A-n55	40412.33	48179.00	19.22%	24666.17	25896.98	4.99%
A-n60	48630.00	60030.80	23.44%	26775.05	28367.96	5.95%
A-n61	43591.08	56808.85	30.32%	26961.10	28675.61	6.36%
A-n62	51956.62	68877.89	32.57%	28001.32	29387.51	4.95%
A-n63a	45283.56	53091.22	17.24%	28007.06	29674.27	5.95%
A-n63b	49717.94	63779.66	28.28%	27768.16	29665.98	6.83%
A-n64	54070.95	61950.68	14.57%	28445.89	29807.02	4.78%
A-n65	55618.02	64036.89	15.14%	28557.17	30065.06	5.28%
A-n69	65905.74	77265.45	17.24%	30769.43	32569.87	5.85%
A-n80	82275.51	102022.93	24.00%	35382.40	37564.58	6.17%
B-n31	13389.95	13817.34	3.19%	14133.60	14947.89	5.76%
B-n34	17466.92	17765.46	1.71%	16745.55	17268.76	3.12%
B-n35	18381.76	22720.89	23.61%	16449.62	16879.40	2.61%
B-n38	18515.91	20030.44	8.18%	17375.05	18035.90	3.80%
B-n39	19623.89	20371.05	3.81%	17638.49	18273.27	3.60%
B-n41	19492.17	24090.47	23.59%	18207.22	18852.89	3.55%
B-n43	20155.06	22099.24	9.65%	19082.90	20448.62	7.16%
B-n44	20867.43	21675.80	3.87%	20216.32	21310.97	5.41%
B-n45a	30040.79	36908.46	22.86%	20659.33	21401.30	3.59%
B-n45b	19892.90	24847.73	24.91%	19755.17	20912.14	5.86%
B-n50a	27220.90	33676.77	23.72%	22820.89	23500.30	2.98%
B-n50b	24760.11	48931.65	97.62%	22734.49	23981.21	5.48%
B-n51	29989.25	36535.76	21.83%	22736.16	23767.84	4.54%
B-n52	28861.21	29260.05	1.38%	22907.44	24317.33	6.15%
B-n56	31408.23	37562.65	19.59%	24372.43	26094.97	7.07%
B-n57a	34807.16	40413.16	16.11%	25626.12	27202.34	6.15%
B-n57b	31897.35	36523.49	14.50%	24967.39	26312.74	5.39%
B-n63	36220.14	43938.83	21.31%	27076.65	29866.52	10.30%
B-n64	32028.25	39831.39	24.36%	27823.53	30300.12	8.90%
B-n66	35485.08	42405.68	19.50%	28156.51	31246.00	10.97%
B-n67	38217.25	48963.71	28.12%	28886.33	31210.35	8.05%
B-n68	39075.71	40794.93	4.40%	29467.00	31316.89	6.28%
B-n78	55145.26	77260.49	40.10%	33804.21	35717.13	5.66%
Average	-	-	21.04%	-	-	4.98%

In this paper, we use the budget uncertainty set to describe the uncertain demand. Compare to the box uncertainty set, the budget uncertainty set can overcome the solution over-conservativeness. Table 3 shows the logistic cost and replenishment quantity gaps between budget and box uncertainty sets, where the baseline is the budget uncertainty set. Using budget uncertainty sets can save 21.12% of the cost than using box uncertainty sets. Because the box uncertainty set is too conservative, the depot will provide more supplies to the shelter to avoid the shortage in extreme cases.

**Table 3:** Logistic cost and replenishment quantity gaps between budget and box uncertainty sets.

Instance	Logistic cost			Replenishment quantity		
	Budget	Box	Gap	Budget	Box	Gap
A-n32	13830.55	16667.75	20.51%	1046.22	1124.91	7.52%
A-n33a	13929.27	16727.25	20.09%	913.22	1058.36	15.89%
A-n33b	14331.09	17198.82	20.01%	968.04	1088.45	12.44%
A-n34	14498.00	17484.35	20.60%	928.59	1131.91	21.90%
A-n36	15200.69	18302.62	20.41%	1105.14	1265.27	14.49%
A-n37a	15915.37	19405.32	21.93%	1304.63	1422.45	9.03%
A-n37b	16366.33	19822.25	21.12%	1138.57	1337.27	17.45%
A-n38	15948.29	19581.10	22.78%	1225.89	1486.64	21.27%
A-n39a	16806.88	20385.52	21.29%	1241.09	1577.73	27.12%
A-n39b	17387.01	20853.85	19.94%	1373.58	1485.18	8.12%
A-n44	18620.07	22686.38	21.84%	1471.35	1676.36	13.93%
A-n45a	19133.85	22935.55	19.87%	1412.45	1807.82	27.99%
A-n45b	18887.41	22980.46	21.67%	1500.13	1708.00	13.86%
A-n46	19320.84	23424.82	21.24%	1570.00	1715.45	9.26%
A-n48	20251.86	24642.99	21.68%	1648.77	2086.36	26.54%
A-n53	22218.30	26977.30	21.42%	1810.52	2220.18	22.63%
A-n54	22536.00	27606.60	22.50%	1947.14	2502.45	28.52%
A-n55	23249.51	27790.60	19.53%	1774.70	2081.45	17.28%
A-n60	25203.82	30260.43	20.06%	2002.88	2638.91	31.76%
A-n61	25249.92	30352.32	20.21%	1922.68	2159.18	12.30%
A-n62	25792.08	31334.63	21.49%	2421.84	2764.55	14.15%
A-n63a	26103.63	31609.87	21.09%	2006.03	2611.18	30.17%
A-n63b	25760.94	31134.26	20.86%	1985.08	2441.91	23.01%
A-n64	26514.35	31890.75	20.28%	2281.98	2862.45	25.44%
A-n65	26380.00	32139.21	21.83%	2222.12	2523.36	13.56%
A-n69	28476.25	34285.42	20.40%	2465.34	2947.73	19.57%
A-n80	32616.40	39549.58	21.26%	3071.44	3301.18	7.48%
B-n31	11815.13	14388.81	21.78%	931.02	1104.18	18.60%
B-n34	14300.57	17004.35	18.91%	990.28	1152.55	16.39%
B-n35	14336.08	17452.08	21.74%	1153.14	1164.91	1.02%
B-n38	14844.98	18017.86	21.37%	1075.92	1161.27	7.93%
B-n39	14941.74	18200.74	21.81%	1209.09	1304.55	7.89%
B-n41	15996.34	19478.40	21.77%	1099.08	1283.82	16.81%
B-n43	16471.69	20022.08	21.55%	1237.56	1373.36	10.97%
B-n44	17401.27	21019.01	20.79%	1202.98	1354.91	12.63%
B-n45a	18178.73	21956.76	20.78%	1517.28	1645.73	8.47%
B-n45b	16846.26	20488.32	21.62%	1268.96	1474.00	16.16%
B-n50a	19888.49	23891.82	20.13%	1468.91	1892.09	28.81%
B-n50b	19857.79	24027.79	21.00%	1321.57	1730.73	30.96%
B-n51	20813.25	25010.43	20.17%	1855.40	1932.64	4.16%
B-n52	19794.28	23927.88	20.88%	1537.92	1730.73	12.54%
B-n56	21057.67	25803.37	22.54%	1845.14	2221.73	20.41%
B-n57a	22323.83	27269.12	22.15%	1666.80	1996.00	19.75%
B-n57b	21864.39	26795.75	22.55%	1470.11	1725.36	17.36%
B-n63	24619.95	29545.08	20.00%	1757.17	1914.18	8.94%
B-n64	24225.35	29448.61	21.56%	1791.66	2165.36	20.86%
B-n66	24593.59	30285.76	23.14%	1967.14	2374.18	20.69%
B-n67	25588.89	31362.87	22.56%	2048.52	2381.36	16.25%
B-n68	25654.70	31353.53	22.21%	2206.04	2463.73	11.68%
B-n78	30695.47	36539.30	19.04%	2733.99	3212.36	17.50%
Average	-	-	21.12%	-	-	16.79%

Furthermore, we analyze the inventory-routing policy on instances A-n32 and B-n78, where the nodes in instance A-n32 is are uniformly distributed and the nodes in instance A-n32 is are clustered. For the small instance A-n32, the truck has the ability to service all shelters in the planning horizon. On the contrary, the instance B-n78 is big enough to simulate the situation that some shelters cannot be serviced by land replenishment mode. For humanitarian considerations, we did not analyze the situation that some shelters must be abandoned due to time inability in dual replenishment mode on very-large-scale instances. Usually, additional working time and vehicles can support these shelters.

**Fig 5:**
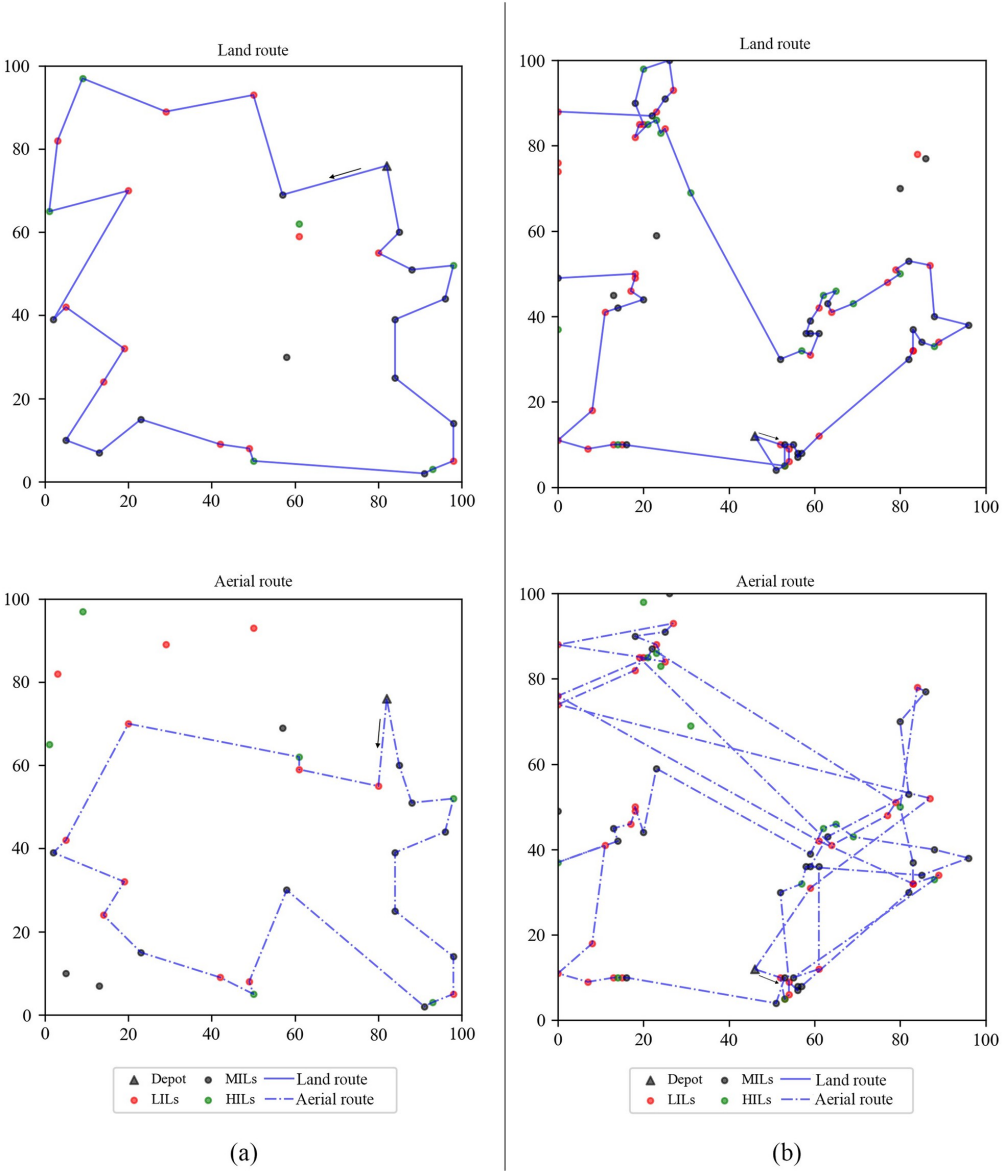
Routing decisions. (a) Routing decisions on A-n32. (b) Routing decisions on B-n78.

Fig 5 shows the best route solved by the proposed ALNS algorithm. Where shelters with a low initial inventory level (LILs with 0 ≤ *I*_0_ ≤ 10) are marked as red points, with a middle initial inventory level (MILs, with 10 < *I*_0_ ≤ 20) are marked as black points, with a high initial inventory level (HILs, with 20 < *I*_0_ ≤ 30) are marked as green points, and the central depot is marked as a black triangle. Results show that on the small-scale instance A-n32, most shelters are replenished by the land replenishment mode and the aerial replenishment mode only as a means of quickly replenishing ways. On the large-scale instance B-n78, most shelters are replenished by helicopter. Some shelters far from the depot are abandoned by the land replenishment mode. In both instances, almost all LILs are replenished with priority and considered the distance factor.

### 5.3 Sensitivity analysis

The two most important parameters that affect the performance of ALNS are the removal rate *ρ* and the reaction factor *η*. We now analyze the value selection of these two parameters based on tuning phase solutions. The removal rate *ρ* is selected from 10%, 15%, 20%, 25%, and 30%. The reaction factor *η* is selected from 0.1, 0.15, 0.2, 0.25, 0.3. We use each combination to test instances A-n32 and B-78 for comparing the best objective value. Tables 4 and 5 shows the logistic cost of the ALNS algorithm with different removal rates and reaction factor tests on instance A-n32 and B-n78, respectively. The results validate the parameter settings we got during the tuning phase that setting the removal rate *ρ* = 20% and the reaction factor *η* = 0.2 have a better performance.

**Table 4:** Logistic cost on instance A-n32 with different removal rate and reaction factor.

*ρ*	0.1	0.15	0.2	0.25	0.3
*η*					
10%	13909.06	13908.43	13942.81	13953.35	13963.15
15%	13968.93	13919.80	13938.90	13913.83	13934.94
20%	13946.24	13944.92	13900.73*	13931.55	13988.52
25%	13974.01	13978.20	13935.70	14029.84	13973.60
30%	14030.41	13971.91	14039.81	13999.10	13981.13

Minimum cost is marked with *

**Table 5:** Logistic cost on instance B-n78 with different removal rate and reaction factor.

*ρ*	0.1	0.15	0.2	0.25	0.3
*η*					
10%	30816.21	30316.17	29928.08	30189.50	30476.23
15%	30557.88	30194.09	29978.30	30133.10	30338.50
20%	30026.90	30057.00	29867.25*	30283.12	30504.71
25%	30552.57	30483.08	29890.69	30325.74	30531.11
30%	31072.91	30310.20	29951.86	30547.84	30795.06

Minimum cost is marked with *

### 5.4 Performance of operators

**Fig 6:**
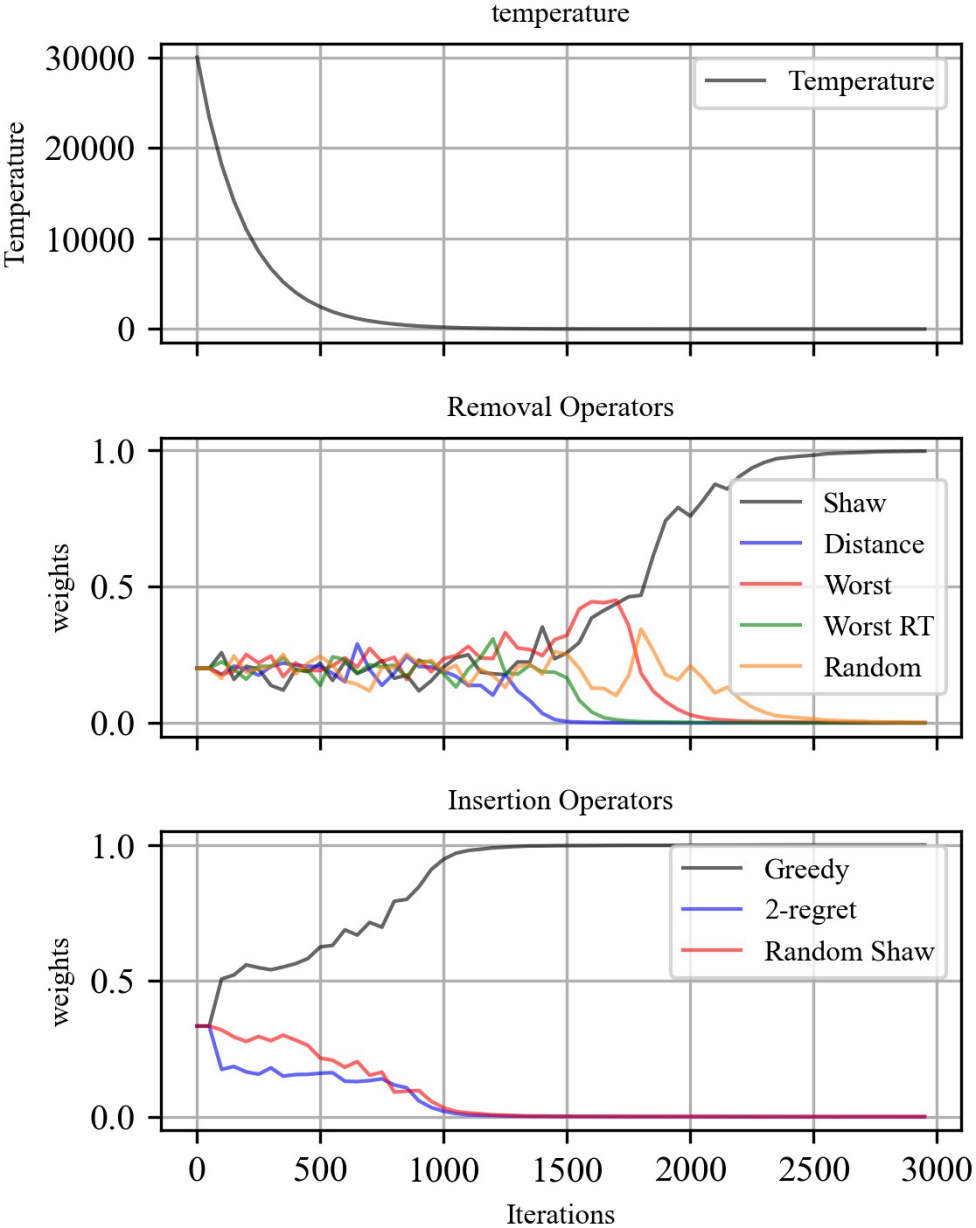
Weight of removal/insertion operators in 3000 iterations on instance A-n32.

We study the computational performance of all operators. Generally speaking, the weights of operators reflect the ability of an operator to find an acceptable/better solution than the current/best solution. Fig 6 shows the weight of different operators in 3000 iterations on instance A-n32. For removal operators, it is easier to accept the current solution when the temperature is high, which means operators get scores more easily. So that weights are not significantly different in the early stage. When the temperature is low, only finding a better solution can have scored, so the weights begin to difference obvious. The Shaw removal operator outperforms other operators in obtaining better solutions, and the distance removal operator performs the worst. For insertion operators, the greedy insertion operator is significantly better than 2-regret and random Shaw from the start.

However, both the new accepted solution and the new better solution are rewarded in the adaptive mechanism. The highest weight operator may not have a better performance for getting the new best solution. We use %IBest/%Usage to measure operators’ performance for getting a new best solution, where %IBest is the percentage of the best solution updated times of an operator to the total best solution updated times, and %Usage is the percentage of an operator used times to the iteration times. Table 6 shows which operator is more likely to update the best solution.

**Table 6:** Performance of operators (%IBest/%Usage).

Instance	Removal Operators				Insertion Operators			
	Shaw	Distance	Worst	Worst RT	Random	Greedy	2-Regret	Shaw
A-n32	2.62	0.62	0.52	1.54	1.06	1.19	0.00	0.00
A-n33a	0.49	0.80	0.00	3.07	1.28	1.09	0.00	0.86
A-n33b	1.56	0.64	1.86	0.94	0.42	1.17	0.00	0.43
A-n34	0.48	1.49	1.24	0.69	1.42	1.15	0.00	0.44
A-n36	2.98	0.36	2.30	0.67	0.42	1.10	0.00	0.76
A-n37a	1.25	1.06	0.40	1.67	1.03	1.07	0.00	0.96
A-n37b	0.88	1.05	1.95	0.59	1.14	1.05	0.96	0.59
A-n38	0.00	2.05	2.32	0.50	0.68	1.07	1.75	0.00
A-n39a	1.53	0.62	0.60	0.95	1.19	1.22	0.00	0.00
A-n39b	2.47	0.00	1.46	0.71	0.56	0.98	2.56	0.00
A-n44	0.22	1.16	1.81	1.25	0.94	1.09	0.75	0.48
A-n45a	1.01	2.92	1.25	0.65	0.60	1.21	0.00	0.00
A-n45b	0.38	0.94	1.91	0.78	1.64	1.20	0.00	0.00
A-n46	0.51	0.69	1.60	0.95	1.20	1.17	0.56	0.00
A-n48	0.62	0.78	0.57	1.40	1.32	1.22	0.00	0.00
A-n53	1.13	1.77	0.57	1.16	0.89	0.97	0.68	1.45
A-n54	1.97	0.73	0.23	1.23	1.30	1.12	0.00	0.71
A-n55	0.73	1.08	0.45	0.52	2.04	1.14	0.00	0.37
A-n60	1.94	0.32	0.38	1.02	1.46	1.19	0.00	0.00
A-n61	0.90	0.88	0.77	0.82	1.71	1.15	0.47	0.00
A-n62	0.46	0.00	0.99	1.20	1.43	1.10	0.00	0.76
A-n63a	1.00	1.01	0.54	0.40	1.95	1.16	0.00	0.34
A-n63b	1.48	0.67	1.13	0.72	0.97	1.19	0.00	0.00
A-n64	0.44	1.77	1.47	1.61	0.56	1.13	0.00	0.44
A-n65	1.61	0.77	0.68	1.76	0.61	1.12	0.00	0.41
A-n69	0.32	0.63	1.27	0.66	1.57	1.18	0.00	0.34
A-n80	0.42	0.88	0.46	0.93	2.24	1.25	0.00	0.46
B-n31	0.70	1.05	0.54	1.03	1.72	1.20	0.00	0.00
B-n34	1.61	1.11	0.84	1.02	0.73	1.17	0.00	0.00
B-n35	1.21	0.81	1.27	0.99	0.83	1.15	0.77	0.00
B-n38	0.41	2.62	1.79	0.00	0.38	0.96	0.00	2.10
B-n39	2.07	1.24	1.08	1.55	0.31	1.21	0.00	0.00
B-n41	0.43	1.37	1.31	0.92	1.05	1.22	0.00	0.00
B-n43	3.34	0.00	0.75	0.48	1.71	1.05	0.00	1.16
B-n44	1.03	0.83	0.78	2.19	0.75	1.12	0.97	0.00
B-n45a	0.61	0.91	1.57	2.54	0.43	1.19	0.00	0.00
B-n45b	0.37	0.00	1.06	0.88	1.97	1.12	0.00	0.61
B-n50a	1.38	0.00	0.79	0.93	1.25	1.12	0.00	0.42
B-n50b	1.25	0.39	2.19	1.20	0.74	1.20	0.00	0.00
B-n51	0.88	1.17	1.66	3.10	0.22	1.20	0.00	0.00
B-n52	1.00	0.98	0.94	1.37	0.51	1.18	0.00	0.00
B-n56	0.59	1.15	1.66	0.64	1.14	1.07	1.43	0.00
B-n57a	0.25	1.23	0.78	1.93	1.49	1.11	0.00	0.58
B-n57b	1.15	0.59	0.86	0.69	1.20	1.13	0.00	0.56
B-n63	0.69	0.78	0.98	1.06	1.16	1.09	0.00	0.77
B-n64	2.18	0.37	0.71	1.16	1.14	1.15	0.00	0.00
B-n66	0.45	0.66	1.07	1.01	1.24	1.15	0.45	0.00
B-n67	0.38	1.54	0.53	0.89	1.34	1.14	0.00	0.44
B-n68	0.80	1.95	0.69	0.00	1.71	1.19	0.00	0.00
B-n78	1.02	1.63	0.75	1.21	1.10	1.05	0.00	1.21
Average	1.06	0.96	1.07	1.10	1.12	1.14	0.23	0.35

For removal operators, Shaw and random removal operators have a better performance, but the difference between each removal operator is insignificant. On the other hand, for insertion operators, the greedy insertion operator significantly outperforms the others. However, it does not mean that worse-performance operators are unnecessary. Table 7 shows the performance of ALNS without one operator (short for no*). The baseline is ALNS.

**Table 7:** Performance of ALNS without * operator (Gaps).

Instance	ALNS	Greedy	2-Regret	Shaw	2-opt
A-n32	13830.55	8.82%	0.32%	0.66%	1.32%
A-n33a	13929.27	10.21%	0.01%	0.00%	0.09%
A-n33b	14331.09	8.39%	0.16%	0.06%	1.41%
A-n34	14498.00	10.47%	1.23%	1.32%	0.91%
A-n36	15200.69	9.77%	0.22%	0.98%	0.78%
A-n37a	15915.37	10.33%	0.64%	0.57%	0.34%
A-n37b	16366.33	8.41%	1.44%	0.81%	1.49%
A-n38	15948.29	10.41%	1.32%	0.18%	1.24%
A-n39a	16806.88	9.14%	0.61%	0.95%	0.29%
A-n39b	17387.01	6.95%	0.08%	0.24%	0.00%
A-n44	18620.07	8.33%	1.19%	0.94%	0.85%
A-n45a	19133.85	10.91%	1.47%	0.46%	1.67%
A-n45b	18887.41	8.69%	1.01%	1.28%	1.37%
A-n46	19320.84	8.08%	0.32%	0.45%	0.38%
A-n48	20251.86	7.00%	0.42%	0.34%	0.71%
A-n53	22218.30	9.55%	0.13%	0.84%	0.32%
A-n54	22536.00	12.08%	1.62%	0.09%	0.93%
A-n55	23249.51	10.33%	0.13%	0.09%	0.20%
A-n60	25203.82	10.37%	0.01%	0.06%	0.29%
A-n61	25249.92	7.93%	0.60%	0.30%	0.23%
A-n62	25792.08	12.55%	0.27%	0.21%	0.38%
A-n63a	26103.63	9.64%	1.17%	0.41%	0.46%
A-n63b	25760.94	10.88%	0.94%	1.33%	0.62%
A-n64	26514.35	11.24%	0.18%	0.03%	0.33%
A-n65	26380.00	11.75%	1.06%	0.87%	1.09%
A-n69	28476.25	10.84%	0.28%	0.27%	0.48%
A-n80	32616.40	12.98%	1.59%	1.82%	1.76%
B-n31	11815.13	9.57%	0.70%	0.80%	0.70%
B-n34	14300.57	17.12%	0.31%	0.00%	0.79%
B-n35	14336.08	14.90%	0.29%	0.30%	0.31%
B-n38	14844.98	12.65%	0.25%	0.08%	0.63%
B-n39	14941.74	16.25%	0.13%	0.25%	0.06%
B-n41	15996.34	12.70%	0.48%	0.49%	0.51%
B-n43	16471.69	15.37%	0.18%	0.31%	0.37%
B-n44	17401.27	15.42%	0.68%	0.49%	0.26%
B-n45a	18178.73	15.95%	0.49%	0.08%	0.05%
B-n45b	16846.26	11.44%	0.04%	0.04%	0.04%
B-n50a	19888.49	7.48%	0.31%	0.18%	0.48%
B-n50b	19857.79	14.69%	0.28%	0.04%	0.39%
B-n51	20813.25	8.75%	0.74%	0.70%	0.79%
B-n52	19794.28	15.29%	0.14%	0.24%	0.30%
B-n56	21057.67	17.27%	0.09%	0.12%	0.93%
B-n57a	22323.83	15.39%	0.02%	0.83%	1.92%
B-n57b	21864.39	14.16%	2.30%	0.48%	1.36%
B-n63	24619.95	9.98%	0.01%	0.06%	0.63%
B-n64	24225.35	15.84%	1.90%	0.41%	0.56%
B-n66	24593.59	17.02%	0.36%	0.12%	0.04%
B-n67	25588.89	16.60%	0.09%	0.37%	0.11%
B-n68	25654.70	17.77%	0.26%	0.35%	0.85%
B-n78	30695.47	11.87%	0.14%	0.31%	0.09%
Average	-	11.79%	0.57%	0.45%	0.64%

It shows that the ALNS with all operators has a better objective value, although fewer operators reduce computation time. This is because although 2-regret and random Shaw insertion operators have insufficient ability to find new optimal solutions, they contribute to exploration ability. In LNS, finding and accepting sub-optimal solutions helps prevent the algorithm from getting stuck in local optima. The result also shows the periodic post-optimization procedure improves the objective by 0.64% on average.

### 5.5 Comparing ALNS with GA

Genetic algorithm (GA) is also a classic algorithm for solving combinatorial optimization problems. Zheng and Zhou (2019) propose a GA for the robust inventory routing problem [26]. The problem they studied considers the effect of routing decisions on lead time but with only one replenishment mode. We compare the results between ALNS and GA on all instances of the Augerat set in Table 8. The baseline is ALNS.

**Table 8:** Comparing ALNS with GA.

Insatance	ALNS	GA	Gap
A-n32	13830.30	15395.17	11.31%
A-n33a	13883.31	15525.40	11.83%
A-n33b	14330.01	15981.08	11.52%
A-n34	14425.55	16224.41	12.47%
A-n36	15199.24	16824.45	10.69%
A-n37a	15926.19	17861.57	12.15%
A-n37b	16315.63	18024.00	10.47%
A-n38	15926.00	17850.67	12.09%
A-n39a	16741.77	18454.52	10.23%
A-n39b	17380.79	18663.96	7.38%
A-n44	18620.77	21530.99	15.63%
A-n45a	18294.56	21132.37	15.51%
A-n45b	18862.77	20892.23	10.76%
A-n46	19253.28	21076.94	9.47%
A-n48	20169.66	22557.06	11.84%
A-n53	22135.84	26300.81	18.82%
A-n54	22506.79	25191.86	11.93%
A-n55	23216.21	25895.65	11.54%
A-n60	25023.56	30121.10	20.37%
A-n61	25255.24	28915.09	14.49%
A-n62	25872.50	31125.06	20.30%
A-n63a	26113.97	29000.71	11.05%
A-n63b	25761.89	32229.04	25.10%
A-n64	26308.90	29591.33	12.48%
A-n65	26329.23	29894.63	13.54%
A-n69	28447.98	36637.29	28.79%
A-n80	32158.70	45618.43	41.85%
B-n31	11791.26	14104.04	19.61%
B-n34	14299.45	17183.95	20.17%
B-n35	14278.29	16426.00	15.04%
B-n38	14854.49	17510.52	17.88%
B-n39	14781.89	17782.22	20.30%
B-n41	15955.40	18563.13	16.34%
B-n43	16618.93	19462.63	17.11%
B-n44	17450.50	20620.08	18.16%
B-n45a	18065.65	21056.47	16.56%
B-n45b	16783.47	20103.48	19.78%
B-n50a	20052.55	23201.03	15.70%
B-n50b	19963.73	23215.09	16.29%
B-n51	20904.29	23070.34	10.36%
B-n52	19748.99	23239.89	17.68%
B-n56	21096.02	25491.32	20.83%
B-n57a	22387.88	26651.96	19.05%
B-n57b	21838.06	25529.38	16.90%
B-n63	24335.06	27679.00	13.74%
B-n64	24438.85	28665.20	17.29%
B-n66	24674.35	28698.74	16.31%
B-n67	25442.03	29768.11	17.00%
B-n68	25671.49	30633.47	19.33%
B-n78	29865.28	38950.17	30.42%
Average	-	-	16.31%

Due to the obvious difference in the efficiency and improvement of the solution in a single iteration of GA and ALNS, for fairness, we only use 1h running time as the termination criteria. The results show that the average performance of ALNS is 16.31% better than that of GA, and ALNS performed better than GA in all instances.

## 6 Conclusions

This paper studies a dual-sourcing inventory routing problem and proposes a robust model. By taking the transportation time as the replenishment lead time, the model considers the problem that the arrival time is highly related to the replenishment quantity in the context of disaster relief. Through air-land transportation, each shelter has two replenishment modes. There are four situations: replenished by truck, by helicopter, by both, and by no replenishment. The mode selection depends on the trade-off between the routing cost and the inventory cost instead of the predetermined. According to the worst case of cumulative demand under the uncertainty set of budget, we derive the closed-form solution of the optimal replenishment quantity. In addition, our optimality conditions do not require any specific uncertainty set. An adaptive large neighborhood search algorithm is developed for the problem.

The numerical experiments examine the proposed model and algorithm on the benchmark instance set. Results show that the logistic cost is effectively reduced when considering the impact of transportation time on lead time. In addition, the dual-sourcing policy is significantly better than only using one replenishment mode. We further test the developed algorithm and show the proposed operators improve the performance of the adaptive large neighborhood search algorithm on the problem. Finally, the comparative experiment validates the proposed algorithm has a better performance than the genetic algorithm.

As with any research, ours begs for further extensions. Considering the impact of routing decisions on replenishment quantity, capacity-constrained multi-vehicle problem is a challenge. Additionally, considering the impact of routing decisions on lead time in a multi-period inventory policy helps address some practical issues in perishables supply chains.

## Supporting information

S1 Appendix Proof of proposition. (DOCX)
