# Supplementary material for: Robust dual sourcing inventory routing optimization for disaster relief
Source: PLoS One. 2023 Apr 27;18(4):e0284971. doi: 10.1371/journal.pone.0284971 (PMC10138234; doi:10.1371/journal.pone.0284971)
Supplement: S1 Appendix — (DOCX) [file pone.0284971.s001.docx]

**S1 Appendix**

**Proof of Proposition.**

The optimal replenishment quantity is balanced between the worst case potential holding costs and shortage costs.

For any shelter $i$, before any replenishment supplies arrive, the inventory cost is

$$\min: \sum_{t=1}^{l_{i}} \max\left\{h(I_{i}-\bar{D}_{it}),s(\underline{D}_{it}-I_{i})\right\}.$$

The inventory costs are not related to the replenishment quantity of each replenishment mode.

It has two situations:

(1) For single replenishment mode, the optimal inventory cost is

$$\min: \sum_{t=l_{i}}^{T} \max\left\{h(I_{i}+q_{i}-\bar{D}_{it}),s(\underline{D}_{it}-I_{i}-q_{i})\right\}.$$

For the holding costs, $h(I_{i}+q_{i}-\bar{D}_{it})$ is a monotonic non-decreasing function, whereas, for the shortage costs, $s(\underline{D}_{it}-I_{i}-q_{i})$ is a monotonic non-increasing function. The optimal replenishment quantity is on one of time point $t_{i}^{*}\in\left\{l,\ldots,T\right\}$, when

$$h(I_{i}+q_{i}-\underline{D}_{it^{*}})=s(\bar{D}_{it^{*}}-I_{i}-q_{i}),$$

which gives

$$q_{i}^{*}=\frac{s\bar{D}_{it^{*}}+h\underline{D}_{it^{*}}}{h+s}-I_{i}.$$

When the right-hand side of is negative for some $t_{i}^{*}$, then the worst case potential holding costs are always more than the worst case potential shortage costs, and the corresponding optimal replenishment quantity is $q_{i}^{*}=0$. Hence, we have the optimality condition for a single replenishment mode of each shelter

$$q_{i}^{*}=\max\left\{\frac{s\bar{D}_{it^{*}}+h\underline{D}_{it^{*}}}{h+s}-I_{i},0\right\}$$

(2) For dual replenishment mode, let $f$ and $s$ represent the first and the second arrival replenishment mode, respectively. The optimal inventory cost is

$$\begin{matrix} \min: \sum_{t=l_{i}^{f}}^{l_{i}^{s}} \max\left\{h(I_{i}+q_{i}^{f}-\bar{D}_{it}),s(\underline{D}_{it}-I_{i}-q_{i}^{f})\right\}+ \\ \sum_{t=l_{i}^{s}}^{T} \max\left\{h(I_{i}+q_{i}^{f}+q_{i}^{s}-\bar{D}_{it}),s(\underline{D}_{it}-I_{i}-q_{i}^{f}-q_{i}^{s})\right\} \end{matrix}$$

Similar with the single replenishment mode, the optimal replenishment quantity of the first arrival replenishment mode is on one of time point $t_{i}^{f*}\in\left\{l_{i}^{f},\ldots,l_{i}^{s}\right\}$, and the optimal replenishment quantity of the second arrival replenishment mode is on one of time point $t_{i}^{s*}\in\left\{l_{i}^{s},\ldots,T\right\}$. Then the optimal replenishment quantities for dual replenishment mode are

$$q_{i}^{f*}=\max\left\{\frac{s\bar{D}_{it^{f*}}+h\underline{D}_{it^{f*}}}{h+s}-I_{i},0\right\},$$

and

$$q_{i}^{s*}=\max\left\{\frac{s\bar{D}_{it^{s*}}+h\underline{D}_{it^{s*}}}{h+s}-q_{i}^{f*}-I_{i},0\right\}.$$
